# Dietary addition of compound organic acids improves the growth performance, carcass trait, and body health of broilers

**DOI:** 10.3389/fnut.2025.1536606

**Published:** 2025-01-28

**Authors:** Fang Cai, Meiping Huang, Wei Liu, Xiaoling Wan, Kai Qiu, Xiao Xu

**Affiliations:** ^1^Hubei Key Laboratory of Animal Nutrition and Feed Science, School of Animal Science and Nutritional Engineering, Wuhan Polytechnic University, Wuhan, China; ^2^Institute of Feed Research, Chinese Academy of Agricultural Sciences, Beijing, China

**Keywords:** broilers, dietary supplementation, compound organic acids, growth characteristics, lipid metabolism

## Abstract

**Introduction:**

The poultry industry constantly seeks strategies to enhance broiler growth performance and overall health. Organic acidifiers, including L-lactic acid, L-malic acid, and acetic acid, have gained attention as potential feed additives to improve animal production by modulating gut health, enhancing nutrient absorption, and supporting immune function. Despite their promising effects in other animal species, the impact of this novel compound organic acidifier on broiler performance, metabolism, and immune response has not been fully elucidated. This study aims to evaluate the effects of this compound acidifier on growth performance, serum lipid profile, antioxidant status, and immune parameters in broilers, providing insights into its potential benefits as a dietary supplement for broiler health and productivity.

**Methods:**

A total of 240 broilers were randomly divided into four groups: a control group and three treatment groups receiving 0.25%, 0.5%, or 1.0% acidifier, with six replicates of ten birds each. Over a 6-week period, various parameters were measured, including serum triglycerides, high-density lipoproteins, lysozyme, immunoglobulins (IgA, IgM), superoxide dismutase (SOD) activity, IL-2, TNF-*α*, and gene expressions related to lipid metabolism.

**Results:**

Over a 6-week period, the acidifier decreased serum triglycerides and high-density lipoproteins while also enhancing growth performance. Additionally, it raised the serum levels of lysozyme, IgA, IgM, and the SOD. Additionally, IL-2 and TNF-*α* concentrations in the jejunum mucosa decreased. The acidifier upregulated *PPARα*, *AMPK*, *FABP1* and *MTTP* expressions, and downregulated *APOB100*. Overall, the acidifier effectively improved broiler growth performance during the early development phase primarily by enhancing hepatic lipid metabolism, antioxidant capacity, and immune function.

**Conclusion:**

These results suggest that the acidifier may accelerate liver lipid metabolism in broilers by modulating the gene expression profiles involved in lipid metabolism.

## 1 Introduction

For many years, it has been common practice to add growth-promoting antibiotics to poultry feed in order to improve profitability. But as a result, microorganisms that are resistant to antibiotics have emerged, which are becoming a growing concern as they can be transmitted from animals to humans (1, 2). As society progresses and the demand for healthier food increases, many countries and regions are now implementing bans or re-strictions on utilizing antibiotics in animal feed and decreasing the reliance on therapeutic antibiotics in livestock and poultry farming (3, 4). Currently, research in the broiler industry is focused on developing new feed additives including organic acids, and bioactive substances as alternatives to antibiotics (5, 6).

Recent studies have increasingly focused on the use of organic acids in broiler feed, recognizing their potential to improve gut health, enhance growth performance, and reduce reliance on antibiotics. Organic acids, such as acetic acid, formic acid, and lactic acid, have been shown to reduce pathogen load in the gastrointestinal tract by lowering pH and inhibiting harmful bacteria, while also promoting the growth of beneficial microbes (7, 8). Additionally, acids like malic acid have demonstrated positive effects on intestinal integrity and antioxidant status in broilers, contributing to improved meat quality (9). Despite these benefits, challenges remain in optimizing the efficacy and stability of organic acids in poultry diets, particularly when used individually or in simple blends. Single acidifiers, though easy to use, often require larger quantities for effectiveness and may be corrosive or unpalatable. In contrast, compound acidifiers, which combine multiple organic acids, offer superior antimicrobial properties and are widely used in the livestock and poultry industries (10). Therefore, introducing a novel compound organic acidifier that combines multiple organic acids in a synergistic manner can effectively address the limitations of single acids.

Acetic acid, a type of short-chain fatty acid, has shown potential for slowing down the aging process in the muscles of older rats (11). It has been proven to be a beneficial dietary treatment for chronic and metabolic diseases (12). L-Malic acid is essential for facilitating the repair of intestinal damage in mice through the polarization of M2 macrophages, which can be achieved through organoid transplantation (13). Tomato seed powder, abundant in malic acid, is an important component for combating free radicals, lowering inflammation, and maintaining a balanced gut microbiome (14). Fermented malic acid has been found to increase the antioxidant levels in broiler muscles and improve the quality of chicken meat (15). Lactic acid supplies energy to cells and functions as a signaling agent that regulates cell activities. Fermented blends of lactic acid-producing bacteria appear to be a promising natural substitute for antibiotics in shielding poultry from *Salmonella* infection (16).

The three organic acids, containing L-malic, L-lactic and acetic acids, are all renewable chemicals used in various industries like food, chemical, pharmaceutical, and cosmetics. By combining these acids to create a new organic acidifier, there is potential for developing feed additives that can improve broiler production. Thus, this study seeks to examine whether and how this novel organic acidifier enhances the broiler performance.

## 2 Materials and methods

### 2.1 Animal experiments

The compound organic acids (COA) were made up of equal parts of L-malic, L-lactic, and acetic acids (in a 1:1:1 ratio), all derived from microbial fermentation. A total of 240 Arbor Acres broilers, weighing 47 ± 3.62 g at hatch, were split into four treatment groups at random, each including six replicates and 10 birds. There were three phases to the 6-week feeding trial: 0 to 2 weeks, 2 to 4 weeks, and 4 to 6 weeks. The normal diet consisting of soybean meal and maize was fed to the control group (Ctrl). Diets containing 0.25, 0.5, and 1.0% of the COA were given to the treatment groups, in that order. The concentrations of COA were selected based on preliminary trials conducted in our laboratory which confirmed that these concentrations provided optimal performance improvements in terms of feed conversion ratio and growth without causing negative impacts on animal welfare or health. The National Research Council’s 1994 guidelines and NY/T 33–2004 were followed in the creation of the basal diet, which was modified for Arbor Acres broilers based on the Feeding Manual (17). All diets were pelleted for feeding.

### 2.2 Animal raising and data collection

Broilers were kept in a three-tier battery system in a controlled environment, where humidity levels were maintained between 50 and 80%, and temperature was carefully regulated to ensure optimal comfort. The light/dark cycle was adjusted to 1 h of darkness and 23 h of light, mimicking natural conditions to support the birds’ circadian rhythm. Each wire cage, measuring 120 × 100 × 48 cm, housed 10 birds, providing sufficient space to allow for natural behaviors and reduce stress. To ensure a comfortable thermal environment, the temperature was initially set at 33°C for the first 3 days and then gradually reduced by 3°C each week, reaching 24°C by the end of the trial.

In addition to meeting the minimum requirements for animal welfare, several additional measures were implemented to further enhance the well-being of the broilers. The birds had unrestricted access to food and clean water at all times, promoting healthy growth and preventing dehydration or malnutrition. Routine health checks were conducted to monitor for any signs of distress, illness, or injury. Mortalities and any abnormalities were recorded daily, and immediate action was taken if any bird showed signs of poor health. Body weight was measured on days 0, 14, 28, and 42 following an 8-h fasting period, and careful attention was paid to minimize any potential discomfort during these procedures. Data on mortality, culling rate, average daily gain, feed intake, and feed conversion ratio were regularly monitored to ensure the overall health and performance of the birds.

### 2.3 Carcass characteristics and sampling

Following an 8-h fast, one broiler each group, roughly weighing the average, was selected for slaughter at the conclusion (day 42) of the trial. After a heart puncture, specimens of blood were obtained and spun at 3000 × g (10 min) at 4°C. The supernatant serum was then stored at −20°C (15). The selected broilers were slaughtered, and their carcass traits were assessed.

The dressing percentage was calculated by dividing the body weight (without blood or feathers) by the broiler’s weight when alive. Half-eviscerated and full-eviscerated per-centages were determined by dividing their respective weights by the broiler’s live weight. Based on the full-eviscerated weight, percentages of abdominal fat, leg muscle, and breast muscle were determined (15).

A cuboidal sample (30 × 15 × 5 mm) was immediately taken from the left-side pectoral muscle post-slaughter. Following the measurement of the muscle sample’s weight and the vertical alignment of the muscle fibers at one end using iron wire, it was sealed in an airtight plastic bag. The bag was then left hanging at 4°C for 24 h. The meat was then carefully excised, and the blotting paper was used to absorb the surface moisture prior to the measurement of drip loss. Additionally, samples of the liver and mid-jejunal mucosa were collected and flash-frozen at −80°C (15).

### 2.4 Quantitative real-time PCR analysis

Total RNA Mini Kit from Magen (Shanghai, China) was used to extract the mRNA from the liver tissue. After that, TaKaRa’s PrimeScriptTM RT reagent Kit (Kyoto, Japan) was applied to obtain cDNA from isolated RNA. Using a TaKaRa RT-qPCR kit, RT-qPCR analysis was carried out on an Analytik Jena AG (Jena, Germany) AJ qTOWER 2.2 Real-Time PCR system. In this work, specific primers that were created on-site and verified for efficacy are mentioned in Table 1. The *GAPDH* was used as the housekeeping gene (18).

**Table 1:** Effects of dietary compound organic acidifier on carcass traits of broilers.

Item, %	Ctrl	COA, %			SEM	*p*- value
		0.25	0.5	1.0		
Dressing percentage	90.39	90.31	90.47	91.75	1.82	0.719
Half evisceration	83.84	82.92	83.76	84.13	1.56	0.387
Full evisceration	77.83	78.24	77.81	77.90	1.04	0.606
Abdominal fat	1.67^a^	1.56^b^	1.49^b^	1.50^b^	0.05	**0.042**
Breast muscle	29.14	29.18	29.47	29.61	0.34	0.708
Leg muscle	29.45	29.43	29.57	29.64	1.77	0.870
Dropping loss	4.89^a^	3.55^b^	3.16^bc^	3.02^c^	0.54	**0.021**

Ctrl, the control group; COA, compound organic acidifier group; SEM, standard error of means. Superscripts in the same row that do not share a small letter indicate a significant difference at *p* < 0.05. Bold values indicate *p* < 0.05.

### 2.5 Serum and Jejunal mucosa analysis

Chemical indices in the serum and jejunal mucosa were analyzed using commercially available kits, following the respective protocols provided by the manufacturers (19). Supplementary Table S1 provided the product codes of these kits. Triglycerides (TG), total cholesterol (TC), very low-density lipoprotein (VLDL), high-density lipoprotein (HDL), immunoglobulin G (IgG), IgM, IgA, total antioxidant capacity (T-AOC), glutathione peroxidase (GSH-PX), and lysozyme were among the parameters measured in serum sample analyses. Secretory immunoglobulin A (sIgA), tumor necrosis factor-*α* (TNF-α), Interleukin-6 (IL-6), IL-2, and were among the indicators measured in the jejunal mucosa.

### 2.6 Statistical analysis

Experimental data were analyzed using one-way ANOVA, followed by Duncan’s multiple comparisons test, with statistical analysis conducted using SAS (v9.1). A significance level of *p* < 0.05 was considered.

## 3 Results

### 3.1 Carcass traits and meat quality

Body weight measurements of broilers were consistent across all experimental groups, as presented in Table 2. Compared to the Ctrl group, broilers fed diets enriched with 0.5% or 1.0% combined organic acids (COA) on days 14, 28, and 42 had considerably greater average body weights. Moreover, broilers supplemented with COA saw a substantial increase in ADG during weeks 1–2, 3–4 and 1–6; however, no differences were seen during weeks 5–6, indicating that COA primarily promotes growth during the first month of broiler development. The experimental treatments had no effect on the broilers’ ADFI. The FCR during weeks 1–2 and weeks 3–4 was decreased with COA supplementation, and during weeks 5–6 and 1–6, it was significantly affected only by the 1.0% COA supplementation. Throughout the feeding trial, the broilers’ daily carcass weight ratio was unaffected by the experimental stimuli.

**Table 2:** Effects of dietary compound organic acidifier on broilers’ development performance.

Item	Ctrl	COA, %			SEM	*p*-value
		0.25	0.5	1.0		
Body weight						
Day 0	43.95	43.93	43.94	43.93	0.04	0.992
Day 14	580.92^b^	579.14^b^	595.50^a^	598.72^a^	11.57	**0.010**
Day 28	1783.99^b^	1804.24^ab^	1838.80^a^	1843.47^a^	43.75	**0.009**
Day 42	3189.36^b^	3227.59 ^ab^	3276.62^a^	3319.43^a^	91.69	**0.043**
Weeks 1 ~ 2						
ADG	38.36^b^	38.23^b^	39.40^a^	39.63^a^	0.83	**0.036**
ADFI	42.89	43.08	42.17	41.90	1.85	0.414
FCR	1.12^a^	1.13^a^	1.07^b^	1.06^b^	0.02	**<0.001**
DCR	0.00	0.00	0.00	0.00	0.00	1.000
Weeks 3 ~ 4						
ADG	85.93^b^	87.51^ab^	88.81^a^	88.91^a^	2.21	**0.041**
ADFI	124.00	123.78	125.85	125.52	3.25	0.399
FCR	1.45^a^	1.42^b^	1.42^b^	1.42^b^	0.03	**0.043**
DCR	0.04	0.03	0.01	0.01	0.04	0.105
Weeks 5–6						
ADG	100.56	101.20	102.90	104.87	5.98	0.177
ADFI	183.01	179.92	184.60	180.32	7.82	0.284
FCR	1.82^a^	1.78^a^	1.81^a^	1.74^b^	0.05	**0.019**
DCR	0.04	0.03	0.01	0.03	0.04	0.769
Weeks 1 ~ 6						
ADG	74.95^b^	75.64^ab^	77.03^a^	77.80^a^	2.08	**0.043**
ADFI	116.63	115.59	117.54	115.91	3.46	0.171
FCR	1.46^a^	1.44^ab^	1.43 ^ab^	1.41^b^	0.04	**0.044**
DCR	0.08	0.06	0.02	0.04	0.05	0.134

Ctrl, the control group; COA, compound organic acidifier; ADG, average daily gain (unit, g); ADFI, average daily feed intake (unit, g); FCR, feed conversion ratio; DCR, death and culling rate (%); SEM, standard error of means. Superscripts in the same row that do not share a small letter indicate a significant difference at *p* < 0.05. Bold values indicate *p* < 0.05.

Table 1 presents the carcass features of broilers. No significant differences were observed between the groups in dressing percentage, half evisceration, full evisceration, or the weights of breast and leg muscles. In contrast to the Ctrl group, the dietary addition of COA produced a notable reduction in the abdomen fat and in the loss of the breast muscle.

### 3.2 Serum indexes and liver genes’ expression related to lipid metabolism

Table 3 demonstrates that, in comparison to the Ctrl group, broilers fed diets with either 0.5% or 1.0% COA had lower levels of serum HDL and TG (*p* < 0.05). Serum levels of VLDL and TC did not, however, differ significantly.

**Table 3:** Effects of dietary compound organic acidifier on serum lipid metabolism of broilers.

Item	Ctrl	COA, %			SEM	*p*- value
		0.25	0.5	1.0		
Total cholesterol, TC	3.24	3.19	3.38	3.15	0.63	0.737
Triglyceride, TG	22.48^a^	21.74^ab^	20.51^b^	18.82^c^	1.26	**0.013**
High-density lipoprotein, HDL	1.37^a^	1.22^ab^	1.16^b^	1.02^c^	0.14	**0.039**
Very-low-density lipoprotein, VLDL	0.62	0.70	0.67	0.75	0.21	0.648

Ctrl, the control group; COA, the compound organic acidifier group; SEM, standard error of means. The unit is mmol/L. Superscripts in the same row that do not share a small letter indicate a significant difference at *p* < 0.05. Bold values indicate *p* < 0.05.

**Figure 1:**
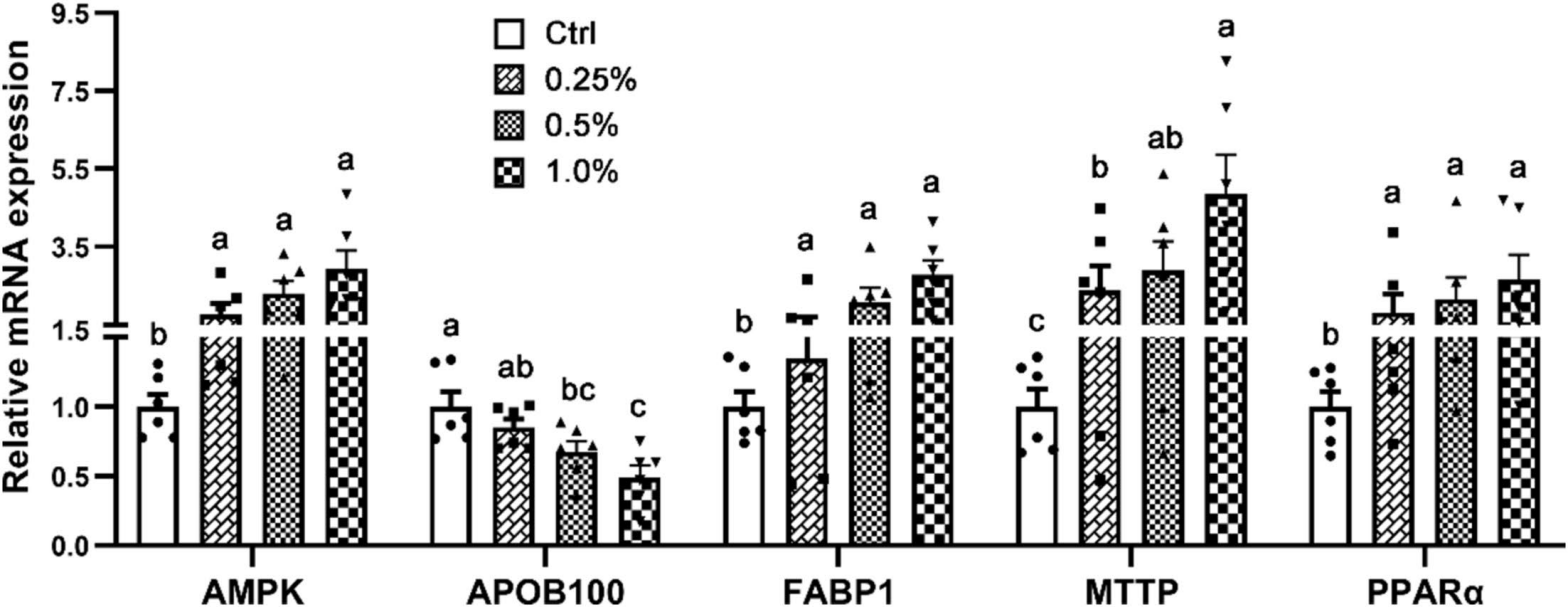
Effects of adding compound organic acidifier to broilers’ diet on the relative mRNA expression of genes controlling hepatic lipid metabolism. Different letters in a panel indicate a significant difference at *p* < 0.05.

Figure 1 shows an analysis of the mRNA profiles of genes connected to the metabolism of lipids in the liver. The genes that were included were *PPARα*, *MTTP*, *FABP1*, *AMPK*, and *APOB100*. In comparison to the Ctrl group, broilers fed diets supplemented with COA exhibited significantly greater levels of *AMPK*, *FABP1*, *MTTP*, and *PPARα*. Furthermore, the treatment group’s liver had much less *APOB100* expressions than the Ctrl.

### 3.3 Antioxidant and immune capacities

The serum and liver’s potential for antioxidants was investigated (Table 4). Broilers given COA exhibited notably greater T-AOC in the liver and serum SOD, and treatment groups with 0.5% or 1.0% COA also showed higher GSH-PX and T-AOC in the serum, and SOD in the liver (*p* < 0.05). There was no discernible difference in the groups’ CAT activity either in the serum or liver. Furthermore, supplementing with dietary COA dramatically reduced the liver’s MDA content (*p* < 0.05). No significant difference was examined across the groups as for the GSH-PX and CAT activities in the liver, and serum MDA.

**Table 4:** Effects of dietary compound organic acidifier on the antioxidant capacity of broilers.

Item	Ctrl	COA, %			SEM	*p*- value
		0.25	0.5	1.0		
Serum						
Catalase, CAT	38.12	36.87	38.46	37.41	5.48	0.679
Superoxide Dismutase, SOD	52.42^c^	59.53^b^	62.74^ab^	66.43^a^	4.19	**0.014**
Glutathione peroxidase, GSH-PX	418.09^b^	434.50^b^	463.71^a^	470.63^a^	32.17	**0.026**
Total anti-oxidative capacity, T-AOC	4.76^c^	5.27^bc^	5.62^ab^	6.58^a^	0.93	**0.034**
Malondialdehyde, MDA	5.73	6.42	5.89	6.65	1.49	0.716
Liver						
Catalase, CAT	43.56	47.34	42.43	49.58	9.82	0.231
Superoxide dismutase, SOD	69.75^c^	73.25^bc^	75.44^ab^	78.96^a^	5.47	**0.032**
Glutathione peroxidase, GSH-PX	318.05	297.54	334.28	340.27	46.84	0.138
Total anti-oxidative capacity, T-AOC	5.69^c^	6.72^b^	8.31^a^	8.46^a^	1.14	**0.027**
Malondialdehyde, MDA	6.80^a^	5.81^b^	5.32^b^	4.35^c^	0.82	**0.045**

Ctrl, the control group; COA, the compound organic acidifier group; CAT, catalase; GSH-PX, glutathione peroxidase; T-AOC, total anti-oxidative capacity; MDA, malondialdehyde; SEM, standard error of means. The unit of CAT, SOD, GSH-PX and T-AOC is U/mL, and the unit of MDA is nmol/mL. Superscripts in the same row that do not share a small letter indicate a significant difference at *p* < 0.05. Bold values indicate *p* < 0.05.

The immunological parameters of broilers are displayed in Table 5. IgA, IgM, and lysozyme levels in the serum significantly increased (*p* < 0.05) when COA was added to the diet, but IgG levels stayed unchanged. With 0.5% or 1.0% COA supplementation, the IL-2 levels in the mucosa of the jejunum considerably reduced compared to the Ctrl. With COA supplementation, there was a substantial drop in TNF-*α* levels in the jejunal mucosa. The levels of secretory immunoglobulin A (sIgA) and IL-6 did not significantly differ between the groups.

**Table 5:** Effects of dietary compound organic acidifier on broilers’ immunity.

Item	Ctrl	COA, %			SEM	*p*- value
		0.25	0.5	1.0		
Serum						
Immunoglobulin A, IgA	1.34^c^	1.47^bc^	1.75^ab^	1.97^a^	0.48	**0.022**
Immunoglobulin M, IgM	1.70^c^	1.86^bc^	2.12^ab^	2.25^a^	0.43	**0.040**
Immunoglobulin G, IgG	3.41	2.98	3.72	3.03	1.32	0.178
Lysozyme	3.84^c^	4.15^c^	4.89^b^	5.45^a^	0.61	**0.011**
Jejunum mucosa						
Interleukin 2, IL-2	6.16^a^	5.80^a^	5.23^b^	4.91^b^	0.65	**0.043**
Interleukin 6, IL-6	1.87	2.34	2.05	1.78	0.76	0.822
Tumor Necrosis Factor-α, TNF-α	2.30^a^	1.85^b^	1.44^c^	1.21^c^	0.52	**0.039**
Secretory immunoglobulin A, sIgA	44.03	36.72	40.37	47.47	13.61	0.307

Ctrl, the control group; COA, the compound organic acidifier group; IgA/M/G, immunoglobulin A/M/G; IL-2/6, interleukin 2/6; TNF-α, tumor necrosis factor-α; sIgA, secretory immunoglobulin A; SEM, standard error of means. IgA/M/G is measured in g/L. Lysozyme is measured in mg/L. IL-2/6 is measured in ng/mL, and sIgA is measured in μg/mL. Superscripts in the same row that do not share a small letter indicate a significant difference at *p* < 0.05. Bold values indicate *p* < 0.05.

## 4 Discussion

Antibiotics are no longer permitted in the production of poultry, so in order to guarantee the best possible growth for the birds, new solutions must be investigated. Using organic acids as feed additives, which have been demonstrated to improve broiler feed intake, growth, and feed efficiency, is one viable substitute.

The organic acidifier offers several unique advantages over existing alternatives to antibiotics in poultry feed. One of its most notable features is its distinct mode of action, which targets pathogenic bacteria by lowering the pH in the gastrointestinal tract, thereby creating an inhospitable environment for harmful microbes while promoting the growth of beneficial bacteria (20). This broad-spectrum efficacy makes it an attractive option for enhancing poultry health and performance. In addition, organic acidifier has been shown to improve feed efficiency without disrupting the delicate balance of the intestinal microbiota, a common issue with other feed additives (21). In contrast, probiotics often face challenges related to their stability, especially under varying environmental conditions, which can limit their effectiveness (22). Similarly, phytobiotics, while promising, show variable efficacy depending on factors such as plant source, dosage, and environmental conditions, making them less reliable in certain settings (23). These limitations underscore the potential of organic acidifier as a more stable and consistent alternative in poultry feed, offering a novel solution to reduce antibiotic use in livestock production. For example, a combination of organic acids has been demonstrated to enhance growth and well-being of broilers suffering from necrotic enteritis (17, 24). Adding mixed organic acids to the diet can increase pancreatic function and enhance the systhesis of tight junction proteins, leading to better broiler output (9, 25). This study confirms the favorable impacts of organic acidifiers on broilers’ performance and carcass quality. The production of broilers may be increased with the help of a compound organic acidifier that combines lactic acid, malic acid, and acetic acid.

The findings related to serum lipid metabolism, as detailed in Table 3, indicate that COA supplementation selectively impacts specific lipid fractions. Notably, reductions in HDL and TG are observed--changes that may indicate a more favorable lipid profile. Given the association between elevated HDL and TG levels with cardiovascular risks, their reduction could suggest improved overall health and meat quality. To elucidate the molecular mechanisms behind these alterations, we analyzed the mRNA expression of key lipid metabolism genes in the liver (Figure 1). Genes such as *PPARα*, *MTTP*, *FABP1*, *AMPK*, and *APOB100*, which regulate lipid uptake, transport, and storage, were analyzed. The increased expression of *AMPK*, a crucial enzyme promoting fatty acid oxidation while inhibiting lipogenesis (26, 27), thus leading to reduced lipid accumulation and improved fat metabolism, supports the hypothesis that COA promotes fatty acid breakdown and reduces lipid accumulation, contributing to the observed reductions in serum TG and HDL levels. Similarly, elevated *PPARα* expression, which regulates fatty acid oxidation (28), further supports this mechanism. Upregulation of *FABP1* and *MTTP* suggests enhanced fatty acid transport and lipoprotein secretion (29, 30), contributing to the observed improved lipid profile. The reduction in *APOB100* expression, a key component of lipoproteins such as VLDL (31), suggests that COA may reduce the synthesis of VLDL particles, which are primarily responsible for the transport of TG from the liver to peripheral tissues. This reduction in *APOB100* expression could explain the lack of significant changes in VLDL and TC levels, as the decrease in VLDL synthesis might be compensated by other lipid transport mechanisms that were not fully captured in the current analysis. Overall, the changes in serum lipid metabolism and the upregulation of key lipid-metabolism-related genes suggest that COA supplementation can enhance lipid processing and promote a healthier lipid profile in broilers. These effects are likely to improve both poultry health and meat quality. While the mRNA profiles provide important insights into the transcriptional regulation of lipid metabolism, it is crucial to confirm the translational impact of these changes by assessing the protein levels of the genes involved. Increased mRNA expression does not always translate into proportional increases in protein levels, as post-transcriptional regulation can influence protein synthesis and stability. Therefore, future studies should investigate the protein expression of related genes to validate the observed mRNA changes and assess the actual protein activity involved in lipid metabolism.

Supplementation with COA significantly improves antioxidant capacity in broilers (Table 4), suggesting a beneficial effect on oxidative stress management. Oxidative stress, caused by an imbalance between reactive oxygen species (ROS) and antioxidant defenses, is a major health concern in poultry (32). Although no significant differences in CAT activity were observed, the increased SOD and GSH-PX activity suggest that COA may protect cellular structures from ROS-induced damage, particularly in the liver and muscle. The reduced liver MDA levels further support COA’s role in mitigating oxidative damage. These findings have important implications for both poultry health and meat quality. Oxidative stress can degrade meat quality by causing lipid oxidation, leading to off-flavors, rancidity, and shorter shelf life. COA supplementation may improve meat quality by reducing oxidative damage, resulting in more stable, flavorful meat with better texture and color. Additionally, enhanced antioxidant status could preserve muscle tissue, potentially extending shelf life by reducing spoilage and oxidative rancidity.

The results in Table 5 show significant changes in immune parameters in broilers supplemented with the COA. These findings suggest that COA may have non-specific effects on immune responses, enhancing both innate and adaptive immunity. The increase in IgA, IgM, and lysozyme points to a strengthened initial defense against pathogens, particularly in the gastrointestinal tract (33, 34). Elevated IgA suggests enhanced mucosal immunity, potentially improving resistance to intestinal infections. The increase in lysozyme further supports enhanced innate immune function. The lack of change in IgG suggests COA’s primary effect on mucosal rather than systemic immunity. The reduction in IL-2 and TNF-*α* indicates potential anti-inflammatory effects, which could help prevent excessive inflammation and support gut health. Further studies are needed to explore the mechanisms of action and evaluate its impact in commercial farming.

COA offers a promising alternative to antibiotics, not only by improving animal health but also through long-term cost savings and environmental sustainability. While COA may have a higher initial cost, it reduces reliance on increasingly expensive and regulated antibiotics, offering savings in healthcare and potentially commanding premium prices by meeting consumer demand for antibiotic-free poultry. Moreover, COA enhances feed efficiency, growth performance, and meat quality, further improving profitability. Environmentally, COA is more sustainable than antibiotics, as it is produced from renewable resources via microbial fermentation, reducing antibiotic use and minimizing environmental contamination from residues and resistance genes. COA’s lower environmental footprint, along with its energy efficiency and reduced waste, supports cleaner, greener poultry farming practices, aligning with the global shift toward sustainable agriculture.

## 5 Conclusion

The recently developed compound organic acidifier, comprising L-malic, L-lactic, and acetic acids, has demonstrated significant benefits in enhancing feed conversion efficiency and growth performance in broilers, particularly during the initial developmental stages. Improvements in immunity, antioxidant capacity, and hepatic lipid metabolism have contributed to better overall performance. The acidifier facilitates lipid metabolism in the liver by upregulating genes involved in fat metabolism (*AMPK*, *FABP1*, *MTTP*, and *PPARα*) and downregulating inhibitory genes (*APOB100*). Our findings suggest that increasing the concentration of COA improves growth performance, with a 0.5% concentration yielding optimal benefits, balancing cost-effectiveness and marginal benefit. For future research, we recommend investigating the long-term effects of COA supplementation on broiler health and meat quality, as well as its impact on other production parameters over extended periods. Furthermore, exploring the effects of different organic acidifier combinations and concentrations could provide deeper insights into their potential for improving broiler health and performance at various growth stages.

## Data Availability

The original contributions presented in the study are included in the article/Supplementary material, further inquiries can be directed to the corresponding authors.
