# Supplementary material for: Dietary addition of compound organic acids improves the growth performance, carcass trait, and body health of broilers
Source: Front Nutr. 2025 Jan 28;12:1536606. doi: 10.3389/fnut.2025.1536606 (PMC11810740; doi:10.3389/fnut.2025.1536606)
Supplement: Supplementary file 1 [file Table_1.docx]

**Table S1.** The information of commercial kits used for chemical analysis.

| Index (Full name) | Index (abbreviation) | Product code |
| --- | --- | --- |
| Total cholesterol | TC | A111-1-1 |
| Triglyceride | TG | A110-1-1 |
| High density lipoprotein | HDL | F003-1-1 |
| Very Low density lipoprotein | VLDL | H249-1-1 |
| Catalase | CAT | A007-1-1 |
| Total superoxide dismutase | SOD | A001-3-2 |
| Glutathione peroxidase | GSH-PX | A005-1-2 |
| Total anti-oxidative capacity | T-AOC | A015-1-2 |
| Malondialdehyde | MDA | A003-1-2 |
| Immunoglobulin A | IgA | H108 |
| Immunoglobulin M | IgM | H109 |
| Immunoglobulin G | IgG | H106 |
| Lysozyme | - | A050-1-1 |
| Interleukin-2 | IL-2 | H003-1-1 |
| Interleukin-6 | IL-6 | H007-1-1 |
| Tumor necrosis factor-α | TNF-α | H052-1-2 |
| Secretory immunoglobulin A | sIgA | H108-2-1 |
